# Comment on: Comparison of 1064‐nm Picosecond and Q‐Switched Neodymium‐Doped Yttrium Aluminum Garnet Lasers for Melasma in Asian Women: A Randomized Double‐Blind Split‐Face Trial

**DOI:** 10.1111/jocd.71181

**Published:** 2026-09-02

**Authors:** Maheen Hamid

**Affiliations:** ^1^ Multan Medical and Dental College Multan Pakistan

Comparison of 1064‐nm Picosecond and Q‐Switched Neodymium‐Doped Yttrium Aluminum Garnet Lasers for Melasma in Asian Women: A Randomized Double‐Blind Split‐Face Trial. According to Handel et al., 2014, melasma is an acquired and chronic hypermelanosis consisting of symmetrical brown macules over the sun‐exposed areas of the face, which is heavily influenced by fluctuating systemic hormones, background ultraviolet radiation and individual skin care practices [1]. We commend the authors for employing a prospective, randomized, double‐blinded, split‐face trial design. This methodology essentially uses each participant as his or her own internal control, effectively eliminating the myriad of confounding variables that are often present in parallel‐group cosmetic trials that can mask the primary intervention. However, there are some methodological considerations we would like to suggest to increase the generalizability of the findings.

Firstly, the picosecond protocol added a diffractive lens array handpiece to the standard flat‐beam passes, while the Q‐switched protocol was based only on collimated flat beams. Thus, the original study compared two different treatment protocols (picosecond laser with a diffractive lens array versus conventional Q‐switched Nd:YAG laser treatment), rather than evaluating the effect of pulse duration alone. The laser‐induced optical breakdown created by fractional handpieces creates plasma‐mediated shockwaves that promote dermal remodeling, which may have contributed to the observed outcomes [2]. Secondly, the immediate comfort advantage of the picosecond platform needs to be compared to the selection of parameters for the hemi‐face in the control group. In the case of Asian skin phototypes, the conventional clinical wisdom for low‐fluence toning with a Q‐switched Nd:YAG laser is to use large spot sizes and low fluences to reduce thermal damage [3]. The control hemi‐face was treated with a higher intensity energy spectrum by reducing the spot size. While treatment discomfort is multifactorial and is influenced by pulse characteristics, peak power, treatment endpoints, cooling methods, and interindividual variability, this setting may also be responsible for the increased immediate pain scores reported by the participants.

Finally, we thank the authors for their contribution of important clinical information for the cosmetic dermatology community. Future comparative trials should consider standardizing spot size, fluence, total delivered energy, treatment endpoints, number of passes, and the use (or omission) of fractional handpiece to better isolate the effect of laser pulse duration [4].

## Funding

The author has nothing to report.

## Disclosure

Declaration of use of AI: The author has nothing to report.

## Ethics Statement

The author has nothing to report.

## Conflicts of Interest

The author declares no conflicts of interest.

## Data Availability

The author has nothing to report.
